# Production and Characterization of Highly Thermostable *β*-Glucosidase during the Biodegradation of Methyl Cellulose by* Fusarium oxysporum*

**DOI:** 10.1155/2016/3978124

**Published:** 2016-02-08

**Authors:** Folasade M. Olajuyigbe, Chidinma M. Nlekerem, Olusola A. Ogunyewo

**Affiliations:** Enzyme Biotechnology and Environmental Health Unit, Department of Biochemistry, Federal University of Technology, Akure 340001, Nigeria

## Abstract

Production of *β*-glucosidase from* Fusarium oxysporum* was investigated during degradation of some cellulosic substrates (Avicel, *α*-cellulose, carboxymethyl cellulose (CMC), and methylcellulose). Optimized production of *β*-glucosidase using the cellulosic substrate that supported highest yield of enzyme was examined over 192 h fermentation period and varied pH of 3.0–11.0. The *β*-glucosidase produced was characterized for its suitability for industrial application. Methyl cellulose supported the highest yield of *β*-glucosidase (177.5 U/mg) at pH 6.0 and 30°C at 96 h of fermentation with liberation of 2.121 *μ*mol/mL glucose. The crude enzyme had optimum activity at pH 5.0 and 70°C. The enzyme was stable over broad pH range of 4.0–7.0 with relative residual activity above 60% after 180 min of incubation. *β*-glucosidase demonstrated high thermostability with 83% of its original activity retained at 70°C after 180 min of incubation. The activity of *β*-glucosidase was enhanced by Mn^2+^ and Fe^2+^ with relative activities of 167.67% and 205.56%, respectively, at 5 mM and 360% and 315%, respectively, at 10 mM. The properties shown by *β*-glucosidase suggest suitability of the enzyme for industrial applications in the improvement of hydrolysis of cellulosic compounds into fermentable sugars that can be used in energy generation and biofuel production.

## 1. Introduction

Cellulose is the most abundant global renewable biopolymer and agricultural waste representing about 1.5 × 10^12^ tons of the total annual biomass production in the tropics [1, 2]. Cellulose is considered as one of the most important sources of carbon globally [3, 4].

The value of cellulose as a renewable source of energy has made cellulose hydrolysis the subject of intense research and industrial interest [2]. There has been much research aimed at obtaining new microorganisms capable of producing cellulolytic enzymes with higher specific activities and greater efficiency [5]. Cellulolytic enzymes play important role in natural biodegradation processes in which plant lignocellulosic materials are efficiently degraded by cellulolytic fungi, bacteria, actinomycetes, and protozoa. In industry, these enzymes have found novel applications in the production of fermentable sugars and ethanol, organic acids, detergents, and other chemicals. Cellulases provide a key opportunity for achieving tremendous benefits of biomass utilization [6]. Cellulolytic enzymes are synthesized by a number of microorganisms. Fungi and bacteria are the main natural agents of cellulose degradation [7].

Enzymes involved in biodegradation of lignocellulosic biomass are those of the cellulase system, of which *β*-glucosidase is a constituent [8]. This is because the complete hydrolysis of cellulose to glucose requires this system of enzymes (cellulases) which comprised endoglucanases, exoglucanases (cellobiohydrolases), and *β*-glucosidase. *β*-glucosidase hydrolyses cellobiose by cleaving the *β*- (1–4) linkage in it to generate D-glucose. Thus, *β*-glucosidases allow the cellulolytic enzymes to function more efficiently by producing glucose from cellobiose and reducing cellobiose inhibition [9]. The increased need for a considerable *β*-glucosidase activity, especially in the enzymatic saccharification of cellulose for bioenergy, has strongly stimulated the study of *β*-glucosidase [8].

The *β*-glucosidase family (EC 3.2.1.21) is a widespread group of enzymes that catalyze the hydrolysis of a broad variety of glycosides [10]. While some organisms secrete either endoglucanase or *β*-glucosidase, in other organisms, *β*-glucosidase is either lacking or produced in insufficient quantities [11]. When *β*-glucosidase secretion is low, cellobiose accumulates instead of glucose [12]. Cellobiose accumulation acts as a feedback-inhibitor of cellulose depolymerization by endo- and exoglucanases [13] which is a critical factor in the industrial scale conversion of cellulose to glucose [14]. This situation can be alleviated during industrial scale conversion of cellulosic biomass by exogenous incorporation of *β*-glucosidase enzyme.

Obtaining efficient and thermostable *β*-glucosidase has become the goal of much research worldwide. Enzyme thermostability is essential during the saccharification step because steam is always used to make the substrates more suitable for enzymatic hydrolysis [15]. Thermostable enzymes can be used simultaneously and directly in the saccharification procedure without a precooling process. In view of the intense requirement of thermostable *β*-glucosidase in industrial applications, we investigated in this study the production of *β*-glucosidase by* F. oxysporum* during the biodegradation of cellulose under different submerged fermentation conditions and the biochemical properties of the produced *β*-glucosidase.

## 2. Materials and Methods

### 2.1. Chemicals

Glycine, p-nitrophenyl-*β*-D-glucopyranoside, methyl cellulose, Avicel, hydrochloric acid, sodium acetate, peptone, sodium trioxocarbonate (IV), sodium hydroxide, potassium dihydrogen phosphate, dipotassium hydrogen phosphate, manganese sulphate, magnesium sulphate, mercury chloride, manganese chloride, calcium chloride, iron (II) chloride, iron (III) chloride, bovine serum albumin (BSA), Tris(hydroxymethyl)aminomethane, and D-glucose were products of Sigma-Aldrich (St. Louis, MO, USA). All other chemicals used were of analytical grade.

### 2.2. Microorganism

The microorganism used was a fungus isolated from decaying wood in a selected citrus plantation in Ijare, Ondo State, Southwest Nigeria. This strain was identified as* Fusarium oxysporum* by the Biotechnology Unit of Federal Institute of Industrial Research, Lagos, based on morphological and biochemical methods described by Collins et al. [16]. The fungal strain was maintained on fresh potato dextrose agar (PDA) slants and stored at 4°C.

### 2.3. Inoculum Preparation and Production of *β*-Glucosidase

Inoculum of* Fusarium oxysporum* was prepared by growing a loopful of slant culture in 100 mL culture medium containing glucose (10.0 g/L), ammonium nitrate (2.0 g/L), KH_2_PO_4_ (0.8 g/L), K_2_HPO_4_ (0.2 g/L), MgSO_4_·7H_2_O (0.5 g/L), and yeast extract (2.0 g/L) in a 200 mL conical flask with pH adjusted to 6.0 [17]. The culture was incubated at 37°C for 72 hr at 160 rpm in a shaking incubator (Stuart, UK). The 3-day-old seed culture was used as inoculum for the production media. Seed inoculum of 4 mL (constituting 4% v/v) was transferred into 100 mL sterile production media which contained methyl cellulose (10 g/L), KH_2_PO_4_ (2 g/L), ZnSO_4_·7H_2_O (0.003 g/L), FeSO_4_·7H_2_O (0.005 g/L), MnSO_4_·7H_2_O (0.002 g/L), MgSO_4_·7H_2_O, (0.3 g/L), CaCl_2_ (0.3 g/L), CoCl_2_ (0.002 g/L), (NH_4_)_2_SO_4_ (1.4 g/L), yeast extract (0.1 g/L), urea (0.3 g/L), and peptone (0.25 g/L) at pH 6.0 and was incubated for 192 h. Cell cultures were harvested at 48 h interval by filtration using Whatman filter paper and the filtrate was centrifuged at 10,000 rpm for 30 minutes at 4°C using refrigerated benchtop centrifuge (Eppendorf 5810R). The supernatant was used as source of extracellular enzyme. Amount of glucose (reducing sugar) liberated during the biodegradation period of cellulose was determined using DNS method [18].

### 2.4. Enzyme Assay

*β*-glucosidase activity was determined according to the method described by Wood and Bhat [19], with some modification. One hundred and fifty microliter (150 *μ*L) of enzyme extract was added to 450 *μ*L of 6.67 mM p-nitrophenyl-*β*-D-glucopyranoside in an Eppendorf tube and incubated at 40°C for 30 minutes. The reaction was terminated with the addition of 400 *μ*L of 1 M Na_2_CO_3_ and the absorbance was recorded at 400 nm against blank. One unit of enzyme activity was defined as the amount of enzyme required to liberate 1 *μ*mol of p-nitrophenol under standard assay condition.

### 2.5. Determination of Glucose Concentration Liberated during Degradation of Cellulose

The concentration of glucose (or reducing sugar) liberated in the biodegradation media was determined spectrophotometrically at 48 h intervals over the biodegradation period according to the method described by Miller [18]. The reaction mixture constituted 300 *μ*L of the supernatant and 700 *μ*L of dinitrosalicylic acid (DNSA) solution, which was boiled at 100°C for 5 minutes. This reaction mixture was cooled under water and absorbance was taken at 575 nm. The amount of glucose liberated during the biodegradation period was estimated using glucose standard curve.

### 2.6. Protein Content Determination

Protein concentration was determined by the method of Bradford [20] using bovine serum albumin (BSA) as standard. In the assay, 200 *μ*L of diluted dye reagent was pipetted into 10 *μ*L of sample solution. The mixture was then incubated at room temperature for 15 minutes to allow proper colour development. The absorbance was measured at 595 nm against blank. The specific activity of *β*-glucosidase was expressed as U/mg protein.

### 2.7. Effect of pH on Production of *β*-Glucosidase and Liberation of Glucose during Cellulose Biodegradation

Production of *β*-glucosidase was investigated under varying pH range of 3.0–11.0 over 96 hours of cultivation period by adjusting the submerged fermentation medium into various pH values at 30°C. The optimal pH for *β*-glucosidase production by* F. oxysporum* was determined at the end of the cultivation period by measuring enzyme activity using standard assays. Amount of glucose (reducing sugar) liberated during the biodegradation period was determined as described earlier.

### 2.8. Effect of Different Forms of Cellulosic Substrates on Production of *β*-Glucosidase and Liberation of Glucose during Cellulose Degradation

Some carbon sources were investigated for their effects on production of *β*-glucosidase by* F. oxysporum*. Avicel, *α*-cellulose, and carboxymethyl cellulose were tested at 1% (w/v) at the determined optimal pH for *β*-glucosidase production by* F. oxysporum* where methyl cellulose served as control. Cultures were grown for 96 hours at 160 rpm. *β*-glucosidase production was measured at the end of the cultivation period to determine the carbon sources that supported highest yield of enzyme. Amount of glucose (reducing sugar) liberated during the biodegradation period was determined as described earlier.

### 2.9. Characterization of *β*-Glucosidase from* F. oxysporum*

#### 2.9.1. Effect of pH on *β*-Glucosidase Activity and Stability

Effect of pH on activity of *β*-glucosidase was determined by assaying for enzyme activity from pH 3.0 to 11.0 using 50 mM of various buffers over the pH range [glycine-HCl (pH 3.0-4.0), sodium acetate (pH 5.0-6.0), Tris-HCl (7.0-8.0), and glycine-NaOH (pH 9.0–11.0)] using the standard assay procedure described earlier. The pH stability of *β*-glucosidase was carried out by incubating the crude enzyme solution in relevant buffers of varying pH (3.0–11.0) without substrate for 180 min at 40°C. Residual *β*-glucosidase activity was determined after 180 min of incubation using the standard assay procedure described earlier.

#### 2.9.2. Effect of Temperature on *β*-Glucosidase Activity and Stability

Effect of temperature on activity of crude enzyme was determined by incubating the reaction mixture at temperatures ranging from 20 to 90°C for 15 min. Thereafter, the activity of *β*-glucosidase was measured as described earlier. The thermal stability was determined by incubating the crude *β*-glucosidase at temperatures ranging from 30 to 90°C for 300 min. Aliquots of the enzyme (100 *μ*L) were withdrawn at 30-minute interval and were used to determine its residual activity. The residual activity was calculated in reference to the activity obtained prior to incubation which served as control.

#### 2.9.3. Effect of Metal Ions on *β*-Glucosidase Activity

The effects of divalent metals ions (Ca^2+^, Mg^2+^, Fe^2+^, Mn^2+^, Cu^2+^, and Hg^2+^) on *β*-glucosidase activity were determined by adding 5 mM and 10 mM of each metallic chloride to the reaction mixture. *β*-glucosidase activity was measured using the standard assay procedure at optimum pH and temperature obtained.

## 3. Results and Discussion

### 3.1. Effect of Cultivation Time on Production of *β*-Glucosidase and Liberation of Glucose during Cellulose Degradation

**Figure 1:**
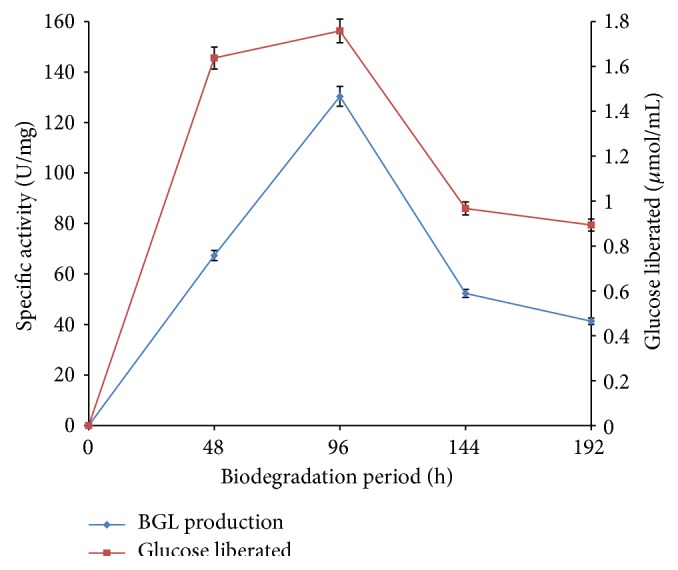
Production of *β*-glucosidase (BGL) and liberation of glucose by* F. oxysporum* over 192 h biodegradation period (error bars represent mean values and standard deviation of triplicate determination).

*β*-glucosidase production was studied to determine the cultivation period for optimal yield of enzyme and its effect on liberation of glucose during the degradation of cellulose. Results obtained for production of *β*-glucosidase and liberation of glucose by* F. oxysporum* are presented in Figure 1. *β*-glucosidase activity was observed at 48 h with specific activity of 67.33 U/mg and increased to a maximum (130 U/mg) at 96 h of cultivation. Similarly, a spontaneous increase in concentration of glucose liberated during the degradation period was observed from the 48 h (1.637 *μ*mol/mL) till the 96 h when maximum liberation of glucose was obtained (1.758 *μ*mol/mL). A sharp decline in production of *β*-glucosidase was observed after 96 h with specific activities of 52.33 U/mg and 41.3 U/mg recorded at 144 h and 192 h, respectively. The decrease in enzyme production consequently affects the hydrolysis of methyl cellulose as reduction in glucose production was likewise obtained after 96 h (Figure 1). Reduction in enzyme production after the optimum cultivation period could be a result of inactivation or inhibition of the fermentation process due to the exhaustion of nutrients in the media or accumulation of toxic wastes that hinders the growth of the fungus [21]. The results obtained suggest a correlation between production of glucose and *β*-glucosidase as the amount of glucose liberated over the cultivation period was dependent on the yield of *β*-glucosidase by* F. oxysporum* [9, 22]. Garcia et al. [23] recently reported an optimum production of *β*-glucosidase from* Lichtheimia ramosa* (27.2 U/mL). However, Quiroz-Castañeda et al. reported maximum production of *β*-glucosidase at 192 h of fermentation by* Bjerkandera adusta* and* Pycnoporus sanguineus* [24].

### 3.2. Effect of pH on *β*-Glucosidase Production and Glucose Liberated during Cellulose Degradation

**Figure 2:**
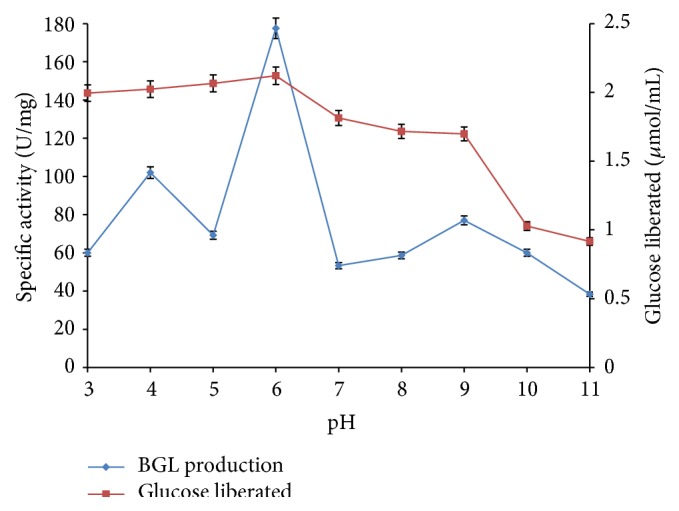
Effects of pH on production of *β*-glucosidase (BGL) and liberation of glucose over 96 h biodegradation period (error bars represent mean values and standard deviation of triplicate determination).

In trying to optimize various conditions that influence enzymatic degradation of cellulose and production of *β*-glucosidase, pH was found to be a critical parameter that affects the process [25]. Production of *β*-glucosidase by* F. oxysporum* increased gradually from pH 3.0 (60 U/mg) till pH 6.0 when optimum production was achieved (177.5 U/mg) as presented in Figure 2. The results revealed a decline in enzyme production after pH 6.0. Similarly, the biodegradation of methyl cellulose by* F. oxysporum* under varying pH conditions revealed that the degradation process increased with pH from pH 3.0 to 6.0 when maximum concentration of liberated glucose was obtained (2.121 *μ*mol/mL). However, after this optimum pH, a gradual decline in the concentration of glucose liberated was observed from pH 7.0 to pH 11.0 (Figure 2). Previous studies have shown that the optimal pH for fungal cellulases varies from species to species [21]. The optimum pH recorded at pH 6.0 in this study supports the findings of Salahuddin et al. and Pérez-Avalos et al., who obtained maximum production of *β*-glucosidase from mesophilic strains of actinomycete and* Cellulomonas flavigena*, respectively, at pH 6.0 [25, 26]. In the same way, Otajevwo and Aluyi reported maximum degradation of cellulose by* Bacillus circulans* at pH 6.0 [27]. However, Fawzi [28] reported optimum production of *β*-glucosidase* Fusarium proliferatum* NRRL26517 at pH 5.0 while Bansal et al. [29] reported pH 7.0 as the optimum which supported maximum production of cellulases by* A. niger* NS-2. Acharya and Chaudhary reported maximum cellulase production by* B. licheniformis* WBS1 (0.388 U/mL) and* Bacillus* WBS3 (0.342 U/mL) at pH values of 8 and 9, respectively [30].

### 3.3. Effect of Carbon Sources on Production of *β*-Glucosidase and Liberation of Glucose during Cellulose Degradation

**Figure 3:**
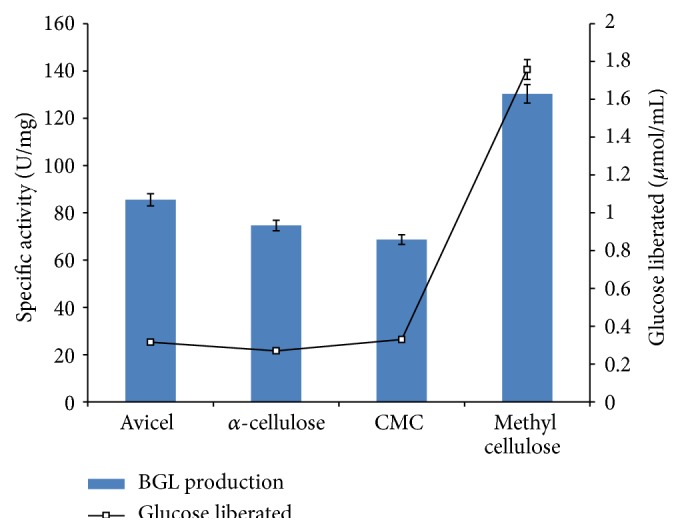
Effects of different forms of cellulose on production of *β*-glucosidase (BGL) and liberation of glucose over 96 h biodegradation period (error bars represent mean values and standard deviation of triplicate determination).

*F. oxysporum* was grown on different commercial cellulosic substrates which include Avicel, *α*-cellulose, carboxymethyl cellulose (CMC), and methyl cellulose (1% w/v) as sole carbon sources to evaluate the substrate that supports optimum yield *β*-glucosidase and liberation of glucose in the degradation process. The results showed that all the commercial substrates tested supported production of *β*-glucosidase by* F. oxysporum* at varying yields with specific activities of 85.5 U/mg, 74.67 U/mg, and 68.67 U/mg obtained with Avicel, CMC, and *α*-cellulose, respectively (Figure 3). Interestingly, methyl cellulose supported the highest yield of *β*-glucosidase (130.33 U/mg; 33.1 U/mL) by* F. oxysporum*. The high yield of *β*-glucosidase recorded with methyl cellulose is remarkable as there has been no report on production of *β*-glucosidase by fungal isolates with methyl cellulose as carbon source. Avicel and CMC have earlier been reported as good substrate for production of *β*-glucosidase [25, 31]. This result therefore makes this form of cellulosic substrate an excellent inducer for the expression of *β*-glucosidase for industrial and biotechnological processes. The moderate production of *β*-glucosidase obtained with Avicel (88.5 U/mg; 16.11 U/mL) when compared with the yield obtained with methyl cellulose (130.33 U/mg) suggests Avicel to also be a good substrate for production of *β*-glucosidase by* F. oxysporum* (Figure 3). However, this result is contrary to some earlier reports where Avicel was reported to adversely affect production of *β*-glucosidase by fungi. Saibi et al. and Sørensen et al. reported low yield of *β*-glucosidase by* Stachybotrys microspora* (0.48 U/mL) and* Aspergillus saccharolyticus* (0.66 U/mL), respectively, when Avicel was used as the carbon source [32, 33].

CMC supported the lowest yield of *β*-glucosidase (68.67 U/mg; 15.12 U/mL) when compared with the yield on other cellulosic substrates tested. The result revealed that the different forms of cellulose used for cultivation affect enzyme production. The low yield of *β*-glucosidase obtained with CMC could be a result of the high viscosity of CMC in the media which limits enzymatic degradation of the cellulose to produce metabolites necessary for growth [34]. However, Salahuddin et al. reported maximum production of *β*-glucosidase from a* mesophilic actinomycete strain KS-22* on CMC [25].

The result for the degradation of different cellulosic substrates tested showed that maximum concentration of glucose liberated during the degradation process was obtained with methyl cellulose (1.76 *μ*mol/mL) and was followed by CMC (0.33 *μ*mol/mL), Avicel (0.31 *μ*mol/mL), and *α*-cellulose (0.21 *μ*mol/mL). The results revealed that the yield of *β*-glucosidase produced by* F. oxysporum* on the different cellulosic substrates is consistent with the amount of reducing sugar (glucose concentration) liberated. However, a striking result was obtained with CMC as a lower yield of *β*-glucosidase was obtained with higher amount of glucose (Figure 3). This could be a result of catabolite repression as production of *β*-glucosidase was repressed in the presence of higher concentration of glucose. Cellulases have been previously reported to be repressed by glucose [32, 34, 35].

### 3.4. Characterization of Crude *β*-Glucosidase from* F. oxysporum*

#### 3.4.1. Effect of pH on Activity and Stability of Crude *β*-Glucosidase

**Figure 4:**
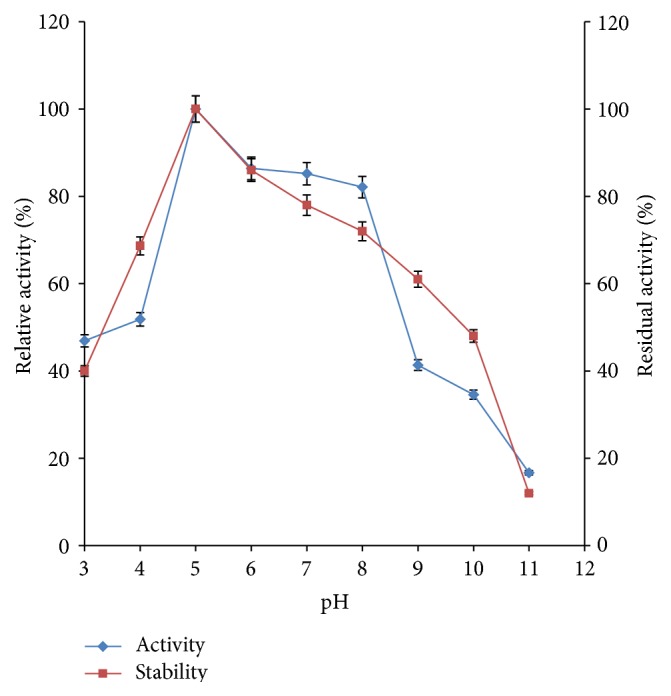
Effect of pH on activity and stability of *β*-glucosidase (error bars represent mean values and standard deviation of triplicate determination).

The effect of pH on *β*-glucosidase activity showed that the enzyme was active over broad pH range of 3.0 to 9.0. An increase in enzyme activity was observed as the pH increased with optimum activity obtained at pH 5.0 after which there was a gradual decline (Figure 4). The result of this study agrees with some earlier reports in which many commercial *β*-glucosidases have been reported to exhibit optimum activity at acidic pH regions. Singhania and Karnchanatat et al. reported similar optimum pH of 5.0 for *β*-glucosidase from* A. niger NII 08121* and* Daldinia eschscholzii*, respectively [36, 37]. *β*-glucosidases from various species of* Penicillium* have been reported to have optimum pH range of 4.0–6.0 [38–40]. Leite et al. obtained maximum production of *β*-glucosidase from* Thermoascus aurantiacus* at pH 4.5 [41]. The enzyme was stable over pH 4.0–9.0 with above 60% of its original activity retained after 180 minutes of incubation (Figure 4). A closely related result has earlier been reported by Kaur et al. that *β*-glucosidase produced by* Melanocarpus *sp. MTCC 3922 was most stable at pH 5.0 at 40°C [42]. These results indicated that the *β*-glucosidase from* Fusarium oxysporum* exhibits a wide range of pH stability and this therefore makes the *β*-glucosidase a good bioresource suitable for use in industrial applications under different pH conditions.

#### 3.4.2. Effect of Temperature on *β*-Glucosidase Activity and Stability

**Figure 5:**
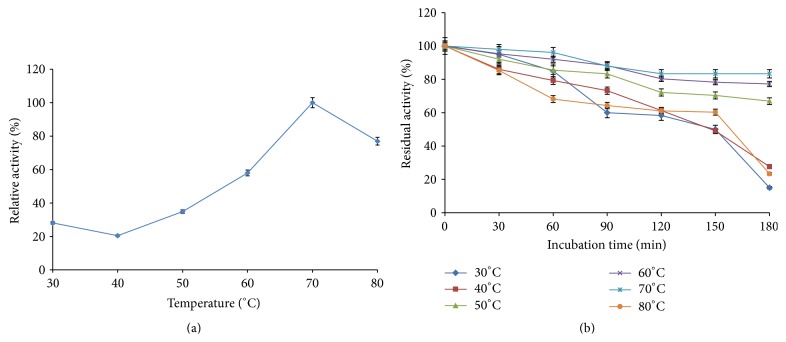
(a) Effect of temperature on *β*-glucosidase activity (error bars represent mean values and standard deviation of triplicate determination). (b) Effect of temperature on *β*-glucosidase (BGL) stability (error bars represent mean values and standard deviation of triplicate determination).

The activity of crude *β*-glucosidase from* F. oxysporum* was determined at different temperatures ranging from 30 to 80°C. The enzyme exhibited optimum activity at 70°C (Figure 5(a)). The result revealed that 57.96% and 77% relative activities were recorded at 60°C and 80°C, respectively (Figure 5(a)). The loss in activity at 80°C temperature could be due to denaturation by heat. The optimum activity of *β*-glucosidase from* F. oxysporum* supports the findings of Singhania [36] who also reported an optimum activity of 70°C for *β*-glucosidase from* A. niger NII 08121* after which the enzyme activity declined. On the contrary, Bhatti et al. reported an optimum temperature and thermostability of 65°C for *β*-glucosidase from* Fusarium solani* [43]. Similarly, an optimum temperature of 60°C has been reported for *β*-glucosidase by species of* Penicillium* genus [39, 40, 44]. The result of the thermostability study showed that *β*-glucosidase from* F. oxysporum* is highly thermostable as it retained above 50% of its original activity after 150 min of incubation at elevated temperatures (Figure 5(b)). The *β*-glucosidase was most stable at 70°C and it exhibited good stability over a wide temperature range of 40°C–80°C. Interestingly, the enzyme retained above 65% of its original activity across this temperature range after 60 min of incubation with 96% residual activity exhibited at 70°C (Figure 5(b)). Many commercial *β*-glucosidases from fungi earlier reported were found to be stable for a short time at high temperatures after which they become denatured [9, 45, 46]. Kaur et al. reported that *β*-glucosidase from* Melanocarpus *sp. MTCC 3922 lost more than 80% of its original activity at 60°C after 30 min of incubation [42]. Liu et al. reported that native *β*-glucosidase secreted by* Aspergillus fumigatus Z5* was moderately stable when incubated for 60 min at temperatures up to 50°C and retained only about 50% of its activity at 70°C [9]. The high stability of this *β*-glucosidase from* F. oxysporum* under prolonged incubation period at high temperatures makes the enzyme thermostable and suitable for use in hydrolysis of cellulosic materials. Thermostable *β*-glucosidases have been reported to exhibit great potential for use in industries such as in food processing and bioconversion of lignocellulolytic biomasses into fermentable sugars for energy generation as they decrease the amount of enzyme needed and remain undenatured under elongated hydrolysis condition [46, 47].

#### 3.4.3. Effect of Metal Ions on *β*-Glucosidase Activity

**Figure 6:**
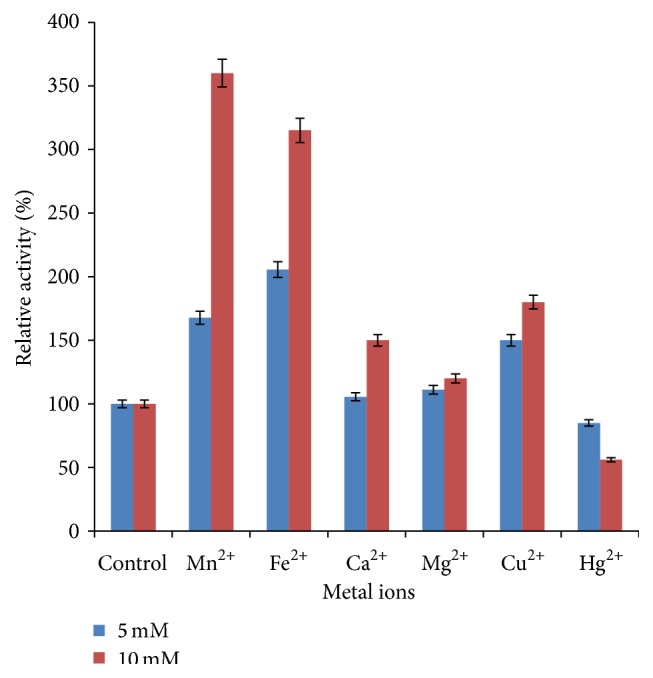
Effect of metal ions on *β*-glucosidase activity (error bar represents mean values and standard deviation of triplicate determination).

The effect of metal ions on the activity of *β*-glucosidase revealed that the activity increased in the presence of all metal ions tested except Hg^2+^. The relative activities obtained in the presence of Mn^2+^, Fe^2+^, Ca^2+^, Mg^2+^, and Cu^2+^ were 167.67%, 205.56%, 105.55%, 111.11%, and 150%, respectively, above the control that was 100% (Figure 6). At higher concentration of 10 mM of the metal ions, a rapid increase in the activity of *β*-glucosidase was observed with relative activities of 360%, 315%, 150%, 120%, and 180% obtained with Mn^2+^, Fe^2+^, Ca^2+^, Mg^2+^, and Cu^2+^, respectively. The increase in enzyme activity in the presence of these metal ions could be due to the response of these ions to certain amino acid residues in the active site of the protein, causing a conformational change in favour of higher activity of the enzyme. *β*-glucosidase activity was inhibited in the presence of Hg^2+^ at both 5 and 10 mM (Figure 6). The results obtained in this study agree with some earlier reports in which *β*-glucosidases of some fungal species were enhanced in the presence of Mn^2+^ and Mg^2+^ [48, 49]. Similarly, Han et al. reported an enhancement in the activity of *β*-glucosidase from* Penicillium simplicissimum* H-11 in the presence of Mn^2+^ and Ca^2+^ [50]. Previous studies have reported an inhibition of *β*-glucosidase activity by Hg^2+^ [44, 51]. A rapid increase in *β*-glucosidase activity in the presence of 10 mM of all the metal ions tested obtained in this study is contrary to the report by Ramanathan et al. that the activity of *β*-glucosidase activity from* F. oxysporum* was inhibited with increase in the concentration of metal ions [52]. The variation could be a result of difference in the strain of the fungus. Bhiri et al. and Han et al. reported an inhibition in the activity of *β*-glucosidase in the presence of Cu^2+^ [44, 50].

## 4. Conclusion

The result from this study revealed methyl cellulose as an excellent substrate for improved production of thermostable *β*-glucosidase from* Fusarium oxysporum* when compared with other forms of cellulose used. The properties shown by *β*-glucosidase suggest the suitability of the enzyme for industrial applications in the hydrolysis of cellulosic compounds into fermentable sugars which can be used in energy generation and biofuel production.

## References

[1] Klemm D., Schmauder H. P., Heinze T. (2002). *Biopolymers*.

[2] Bhat M. K. (2000). Cellulases and related enzymes in biotechnology. *Biotechnology Advances*.

[3] Nowak J., Florek M., Kwiatek W. (2005). Composite structure of wood cells in petrified wood. *Materials Science and Engineering C*.

[4] Sharma S., Vaid S., Bajaj B. J. (2015). Screening of thermo-alkali stable fungal xylanases for potential industrial applications. *Current Research in Microbiology and Biotechnology*.

[5] Subramaniyan S., Prema P. (2000). Cellulase-free xylanases from *Bacillus* and other microorganisms. *FEMS Microbiology Letters*.

[6] Wen Z., Liao W., Chen S. (2005). Production of cellulase by *Trichoderma reesei* from dairy manure. *Bioresource Technology*.

[7] Lederberg J. (1992). Cellulases. *Encyclopaedia of Microbiology*.

[8] Mfombep P. M., Senwo Z. N., Isikhuemhen O. S. (2013). Enzymatic activities and kinetic properties of *β*-glucosidase from selected white rot fungi. *Advances in Biological Chemistry*.

[9] Liu D., Zhang R., Yang X. (2012). Characterization of a thermostable *β*-glucosidase from *Aspergillus fumigatus* Z5, and its functional expression in *Pichia pastoris X33*. *Microbial Cell Factories*.

[10] Berrin J.-G., Czjzek M., Kroon P. A. (2003). Substrate (aglycone) specificity of human cytosolic *β*-glucosidase. *Biochemical Journal*.

[11] Kumar R., Singh S., Singh O. V. (2008). Bioconversion of lignocellulosic biomass: biochemical and molecular perspectives. *Journal of Industrial Microbiology and Biotechnology*.

[12] Stockton B. C., Mitchell D. J., Grohmann K., Himmel M. E. (1991). Optimum beta-D-glucosidase supplementation of cellulase for efficient conversion of cellulose to glucose. *Biotechnology Letters*.

[13] Morais H., Ramos C., Forgacs E., Jakab A., Cserhati T. (2004). Enzyme production of *Pleurotus ostreatus* evaluated by the three-way principal component analysis. *Engineering in Life Sciences*.

[14] Cai Z., Xing G., Yan X. (1997). Methane and nitrous oxide emissions from rice paddy fields as affected by nitrogen fertilisers and water management. *Plant and Soil*.

[15] Liu D., Zhang R., Yang X. (2011). Expression, purification and characterization of two thermostable endoglucanases cloned from a lignocellulosic decomposing fungi *Aspergillus fumigatus* Z5 isolated from compost. *Protein Expression and Purification*.

[16] Collins C. H., Patricia M., Grage J. M. (1991). *Collins and Lynes Microbiological Methods*.

[17] Kachlishvili E., Khardziani T., Metreveli E., Kobakhidze A., Elisashvili V. (2012). Screening of novel basidiomycetes for the production of lignocellulolytic enzymes during fermentation of food wastes. *Journal of Waste Conversion, Bioproducts and Biotechnology*.

[18] Miller G. L. (1959). Use of dinitrosalicylic acid reagent for determination of reducing sugar. *Analytical Chemistry*.

[19] Wood T. M., Bhat K. M., Wood W. A., Kellogg J. A. (1998). Method for measuring cellulase activities. *Methods in Enzymology: Cellulose and Hemicellulose*.

[20] Bradford M. M. (1976). A rapid and sensitive method for the quantitation of microgram quantities of protein utilizing the principle of protein-dye binding. *Analytical Biochemistry*.

[21] Shahzadi T., Anwar Z., Iqbal Z. (2014). Induced production of exoglucanase, and *β*-glucosidase from fungal co-culture of *T. viride* and *G. lucidum*. *Advances in Bioscience and Biotechnology*.

[22] Saha B. C., Freer S. N., Bothast R. J. (1994). Production, purification, and properties of a thermostable *β*-glucosidase from a color variant strain of *Aureobasidium pullulans*. *Applied and Environmental Microbiology*.

[23] Garcia N. F. L., Santos F. R. D. S., Gonçalves F. A., da Paz M. F., Fonseca G. G., Leite R. S. R. (2015). Production of *β*-glucosidase on solid-state fermentation by *Lichtheimia ramosa* in agroindustrial residues: characterization and catalytic properties of the enzymatic extract. *Electronic Journal of Biotechnology*.

[24] Quiroz-Castañeda R. E., Balcázar-López E., Dantán-González E., Martinez A., Folch-Mallol J., Martínez-Anaya C. (2009). Characterization of cellulolytic activities of *Bjerkandera adusta* and *Pycnoporus sanguineus* on solid wheat straw medium. *Electronic Journal of Biotechnology*.

[25] Salahuddin K., Ram P., Suresh G. H., Manish V. D., Virendra S. K., Dilshad H. M. (2012). Biochemical characterization of thermostable cellulose enzyme from mesophilic strains of actinomycete. *African Journal of Biotechnology*.

[26] Pérez-Avalos O., Sánchez-Herrera L. M., Salgado L. M., Ponce-Noyola T. (2008). A bifunctional endoglucanase/endoxylanase from *Cellulomonas flavigena* with potential use in industrial processes at different pH. *Current Microbiology*.

[27] Otajevwo F. D., Aluyi H. S. A. (2011). Cultural conditions necessary for optimal cellulase yield by cellulolytic bacterial organisms as they relate to residual sugars released in broth medium. *Modern Applied Science*.

[28] Fawzi E. M. (2003). Production and purification of *β*-glucosidase and protease by *Fusarium proliferatum* NRRL 26517 grown on *Ficus nitida* wastes. *Annals of Microbiology*.

[29] Bansal N., Tewari R., Soni R., Soni S. K. (2012). Production of cellulases from *Aspergillus niger* NS-2 in solid state fermentation on agricultural and kitchen waste residues. *Waste Management*.

[30] Acharya S., Chaudhary A. (2011). Effect of nutritional and environmental factors on cellulases activity by thermophilic bacteria isolated from hot spring. *Journal of Scientific and Industrial Research*.

[31] Venturi L. L., de Lourdes Polizeli M., Terenzi H. F., dos Prazeres Melo Furriel R., Jorqe J. A. (2002). Extracellular *β*-d-glucosidase from *Chaetomium themophilum var. coprophilum*: production, purification and some biochemical properties. *Journal of Basic Microbiology*.

[32] Saibi W., Abdeljalil S., Gargouri A. (2011). Carbon source directs the differential expression of *β*-glucosidases in *Stachybotrys microspora*. *World Journal of Microbiology and Biotechnology*.

[33] Sørensen A., Andersen J. J., Ahring B. K., Teller P. J., Lübeck M. (2014). Screening of carbon sources for beta-glucosidase production by *Aspergillus saccharolyticus*. *International Biodeterioration and Biodegradation*.

[34] Do B.-C., Dang T.-T., Berrin J.-G. (2009). Cloning, expression in *Pichia pastoris*, and characterization of a thermostable GH5 mannan endo-1,4-*β*-mannosidase from *Aspergillus niger* BK01. *Microbial Cell Factories*.

[35] Suto M., Tomita F. (2001). Induction and catabolite repression mechanisms of cellulase in fungi. *Journal of Bioscience and Bioengineering*.

[36] Singhania R. R. (2011). *Beta-glucosidase from Aspergillus niger NII 08121: “molecular characterization and applications in bioethanol production” [Ph.D. thesis]*.

[37] Karnchanatat A., Petsom A., Sangvanich P. (2007). Purification and biochemical characterization of an extracellular *β*-glucosidase from the wood-decaying fungus *Daldinia eschscholzii* (Ehrenb.:Fr.) Rehm. *FEMS Microbiology Letters*.

[38] Bai H., Wang H., Sun J. (2013). Production, purification and characterization of novel beta glucosidase from newly isolated *Penicillium simplicissimum* H-11 in submerged fermentation. *EXCLI Journal*.

[39] Ramani G., Meera B., Vanitha C., Rao M., Gunasekaran P. (2012). Production, purification, and characterization of a *β*-glucosidase of *Penicillium funiculosum NCL1*. *Applied Biochemistry and Biotechnology*.

[40] Krogh K. B. R. M., Harris P. V., Olsen C. L. (2010). Characterization and kinetic analysis of a thermostable GH3 *β*-glucosidase from *Penicillium brasilianum*. *Applied Microbiology and Biotechnology*.

[41] Leite R. S. R., Gomes E., da Silva R. (2007). Characterization and comparison of thermostability of purified *β*-glucosidases from a mesophilic *Aureobasidium pullulans* and a thermophilic *Thermoascus aurantiacus*. *Process Biochemistry*.

[42] Kaur J., Chadha B. S., Kumar B. A., Saini H. S. (2007). Purification and characterization of two endoglucanases from *Melanocarpus* sp. MTCC 3922. *Bioresource Technology*.

[43] Bhatti H. N., Batool S., Afzal N. (2013). Production and characterization of a novel *β*-glucosidase from *Fusarium solani*. *International Journal of Agriculture and Biology*.

[44] Bhiri F., Chaabouni S. E., Limam F., Ghrir R., Marzouki N. (2008). Purification and biochemical characterization of extracellular *β*-glucosidases from the hypercellulolytic Pol6 mutant of *Penicillium occitanis*. *Applied Biochemistry and Biotechnology*.

[45] Xue Y.-P., Jin L.-Q., Liu Z.-Q., Zhang J.-F., Zheng Y.-G. (2008). Purification and characterization of *β*-glucosidase from *Reticulitermes flaviceps* and its inhibition by valienamine and validamine. *African Journal of Biotechnology*.

[46] Baraldo A., Borges D. G., Tardioli P. W., Farinas C. S. (2014). Characterization of *β*-glucosidase produced by *Aspergillus niger* under solid-state fermentation and partially purified using MANAE-Agarose. *Biotechnology Research International*.

[47] Viikari L., Alapuranen M., Puranen T., Vehmaanperä J., Siika-Aho M. (2007). Thermostable enzymes in lignocellulose hydrolysis. *Biofuels*.

[48] Chen H.-L., Chen Y.-C., Lu M.-Y. J. (2012). A highly efficient *β*-glucosidase from the buffalo rumen fungus *Neocallimastix patriciarum W5*. *Biotechnology for Biofuels*.

[49] Ma S.-J., Leng B., Xu X.-Q. (2011). Purification and characterization of *β*-1,4-glucosidase from *Aspergillus glaucus*. *African Journal of Biotechnology*.

[50] Han X., Hongzhi B., Hui W. (2013). Production, purification and characterization of novel beta-glucosidase from newly isolated *Penicillulm simplicissimum H-11* in submerged fermentation. *Experimental and Clinical Sciences Journal*.

[51] Elshafei A. M., Hassan M. M., Morsi N. M., Elghonamy D. H. (2011). Purification and some kinetic properties of *β*-glucosidase from *Aspergillus terreus NRRL 265*. *African Journal of Biotechnology*.

[52] Ramanathan G., Banupriya S., Abirami D. (2010). Production and optimization of cellulase from *Fusarium oxysporum* by submerged fermentation. *Journal of Scientific and Industrial Research*.

